# Different Aspects of the Neural Response to Socio-Emotional Events Are Related to Instability and Inertia of Emotional Experience in Daily Life: An fMRI-ESM Study

**DOI:** 10.3389/fnhum.2018.00501

**Published:** 2018-12-11

**Authors:** Julian Provenzano, Jojanneke A. Bastiaansen, Philippe Verduyn, Albertine J. Oldehinkel, Philippe Fossati, Peter Kuppens

**Affiliations:** ^1^Faculty of Psychology and Educational Sciences, KU Leuven, Leuven, Belgium; ^2^Interdisciplinary Center Psychopathology and Emotion regulation, Department of Psychiatry, University Medical Center Groningen, University of Groningen, Groningen, Netherlands; ^3^Department of Education and Research, Friesland Mental Health Care Services, Leeuwarden, Netherlands; ^4^Faculty of Psychology and Neuroscience, Maastricht University, Maastricht, Netherlands; ^5^Institut du Cerveau et de la Moelle Epinière, ICM, INSERM U 1127, CNRS, UMR 7225, Sorbonne Université, Paris, France; ^6^AP-HP, Hôpital de la Pitié Salpêtrière, Service de Psychiatrie d’Adultes, Paris, France

**Keywords:** emotion dynamics, emotional inertia, emotional instability, fMRI, salience network, social feedback, ESM

## Abstract

Emotions are fundamentally temporal processes that dynamically change over time. This temporal nature is inherently involved in making emotions adaptive by guiding interactions with our environment. Both the size of emotional changes across time (i.e., emotional instability) and the tendency of emotions to persist across time (i.e., autocorrelation of emotional experience, emotional inertia) are key features of a person’s emotion dynamics, and have been found central to maladaptive functioning and psychopathology as well as linked to social functioning. However, whether different (neural) mechanisms are underlying these dynamics as well as how they are related to the processing of (socio-) emotional information is to date widely unknown. Using a combination of Experience Sampling methods (ESMs) and fMRI (involving a social feedback paradigm), we examine how emotional instability and inertia in everyday life are associated with different aspects of the neural response to socio-emotional events. The findings indicate that while emotional instability is connected to the response of the core salience network (SN), emotional inertia is associated to responses in the parahippocampal gyrus (PHG) and lateral orbitofrontal cortex (lOFC). This is the first study showing that different aspects of the neural response to socio-emotional events are associated with different aspects of the temporal dynamics of emotion in real life.

## Introduction

While defining, describing and measuring emotions remain subject to controversy in emotion research (for example Izard, 2010), there is wide agreement on their core functionality. Indeed, most if not all theories and perspectives on emotions agree, that the basic function of emotions involves informing the organism about relevant changes in the environment and preparing as well as facilitating appropriate responses to such changes (Scherer, 2009; LeDoux, 2012; Adolphs, 2016). This implies that to be adaptive, it is central to the nature of emotions that they—in line with internal and external demands—dynamically change over time (Waugh et al., 2011; Kuppens, 2015).

Such dynamics can be broadly conceptualized as being governed by two opposing tendencies (see Kuppens and Verduyn, 2017). On the one hand, emotions have the tendency to change corresponding to internal and external events, maximizing the fit of the emotional experience with current situational demands. On the other hand, emotions have the tendency to persist, carry on from one moment (situation) to the next by facilitating the perception and construing of the environment congruent to them (Okon-Singer et al., 2015; see also Cunningham et al., 2013). Both tendencies are equally important for the ability of emotions to dynamically and adaptively change, lying at the heart of the adaptive functionality of emotions (Kuppens and Verduyn, 2017). In turn, imbalances between these tendencies could be a major factor in turning emotional experiences maladaptive and interfere with our ability to adaptively interact with our environment.

Accordingly, a recent meta-analysis demonstrated that both the increased tendency to experience unstable emotions (i.e., the magnitude of emotional change from one moment to another; emotional instability) as well as the increased tendency of emotional experiences to endure (i.e., autocorrelation of emotional experience; emotional inertia) in daily life are consistently associated with reduced well-being, maladaptive personality traits like neuroticism and the likelihood of a diagnosis of affective disorder (Kuppens et al., 2010; Houben et al., 2015). While emotional instability is considered a general hallmark of maladjustment and a consistent feature of several affective disorders, inertia is particularly relevant to major depression disorder (MDD), as it is positively associated with severity of depressive symptoms (Koval et al., 2013; Brose et al., 2015), the onset of depressive episodes (Kuppens et al., 2012; van de Leemput et al., 2014), and genetic risk factors of major depression (van Roekel et al., 2018). Furthermore, state-changes in emotion dynamics have been found to be connected to socio-emotional functioning. Fairbairn and Sayette (2013) found that alcohol-induced reduction of negative affect (NA) inertia was mediating improved social functioning and reward effects after alcohol consumption. Such a link between emotion dynamics and social-emotional functioning could be highly relevant, considering its outstanding role in the development and maintenance of affective disorders as depression (Lewinsohn et al., 1999; Monroe et al., 1999).

Thus, understanding how individual differences in emotion dynamics are reflected in the neural mechanisms underlying emotional experience—and in this context especially socio-emotional experiences—could not only be relevant in regard to understanding the organization of emotions themselves, but also inform us about central aspects of affective and mood disorders.

To date, it is widely agreed upon that individual differences in emotion dynamics reflect alterations in the processing of emotional information. Instability is mainly thought to reflect increased sensitivity and reactivity to emotional events (Trull et al., 2015). Inertia has especially been argued to reflect an inflexibility of the emotional response based on inadequate regulatory processes (Hollenstein, 2015). This idea is supported by findings connecting emotional instability with increased emotional reactivity (Thompson et al., 2012), and inertia with decreased recovery from (negative) emotional events leading to sustained emotional experience (Koval et al., 2015). Therefore, these tendencies could reflect mechanisms on different levels of processing of emotional information, determining different aspects of the emotional response.

Interestingly, also on a neural level, it has been proposed that separate mechanisms are underlying emotional reactivity and sustained emotional experience. Cunningham et al. (2013) argue that activation in the salience network (SN)—specifically the amygdala—reflects a fast-initial response to emotional stimuli, while subsequent processes, that allow for more nuanced interpretation and contextualization—involving the orbitofrontal cortex—would, with time, gain increasing importance in the emotional response.

In line with the proposal of different functional networks underlying these tendencies, recent fMRI-studies have connected instability of negative emotions in daily life to reduced resting state functional connectivity between the SN and other subnetworks in remitted depressed subjects (Servaas et al., 2017). Further, negative emotional inertia in daily life is predicted by resting state increase in cerebral blood flow (CBF) in the lateral prefrontal cortex (lPFC) after an emotional task (Waugh et al., 2017).

These findings are especially striking, since altered patterns of intrinsic functional connectivity and alterations in responses of the SN to negative stimuli, have been consistently found in patients suffering from anxiety disorders (e.g., Etkin and Wager, 2007; Sylvester et al., 2012) and depression (e.g., Siegle et al., 2002; Manoliu et al., 2014), healthy participants at risk of affective disorders (e.g., van der Werff et al., 2013), and are generally interpreted as depicting an increased reactivity to (negative) emotional events (e.g., Liberzon and Abelson, 2016). In contrast the lPFC is usually connected to cognitive control (Koechlin et al., 2003) and emotion cognition integration (Gray et al., 2002), and has been proposed to be an important region for emotion regulation (Ochsner et al., 2012).

Research on the neural correlates of emotion dynamics in everyday life has thus far been constrained to resting-state measurements looking into more global between-subject differences in the neural organization of the brain. How daily life emotion dynamics relate to brain processes when people are actually processing and responding to emotional stimuli remains largely unknown. In the present study we aim to directly investigate how the extent of event-related neural responses to socio-emotional stimuli are associated with emotional instability and inertia in daily life, and to verify whether different neural processes underlie these tendencies.

In this study, participants reported their feeling states several times a day over the course of 2 weeks throughout their normal daily lives, and were subsequently confronted with personally relevant positive, neutral and negative social feedback inside the scanner. Valence-general as well as valence-specific event-related neural responses were then associated with between-subject differences in emotional instability and inertia during daily life.

Based on the proposal of Cunningham et al. (2013) and the findings discussed above, we expected emotional instability and emotional inertia to be associated with different aspects of the neural response to emotional stimuli—representing the engagement of different functional networks. We expected emotional instability to be related to an increased activation within SN-regions, particularly involving the amygdala, anterior insula, and dorsal anterior cingulate cortex (dACC). We predicted that emotional inertia would be associated with activations within functional networks that have been associated with evaluative and control processes including the orbitofrontal cortex and regions of the fronto-parietal control network (FPCN).

Emotion measures in daily life were collected using the Experience Sampling Method (ESM), offering a well-established and reliable way to repeatedly obtain ecological valid measures (Csikszentmihalyi and Larson, 1987), which have been extensively used to measure emotion dynamics (see Houben et al., 2015). Since the most robust connection between patterns of emotional experience in everyday life and different indicators for well-being have been found for NA (see Houben et al., 2015) we focused on the dynamics of negative emotional experience.

Socio-emotional events inside the scanner were created using a social feedback task, that has been successfully adapted for fMRI studies (Davey et al., 2011) and allowed us to create events that were both social and self-relevant. These dimensions are especially important since they have been shown to influence how we process emotional information (social: for example Britton et al., 2006; self-relevance: for example Bayer et al., 2017) as well as are a common—if not the most common—feature of emotional events in everyday life (see for example Parkinson, 1996; Tiedens and Leach, 2004; Parkinson et al., 2005; Butler, 2015). Furthermore, this paradigm offers the possibility to investigate both, positive as well as negative events inside the scanner, allowing us to differentiate between valence-specific (only positive or negative) and valence general (positive and negative) associations between socio-emotional events inside the scanner and temporal dynamics of (negative) emotional experience in daily life.

In order to limit the influence of potentially confounding factors and increase the power of estimating true trait-differences in the processing of socio-emotional feedback as well as emotion dynamics in everyday life we chose a very homogeneous sample that was restricted to female students.

## Materials and Methods

### Participants

Increasing the comparability of ESM measurements and limiting the influence of confounding factors, we chose to collect a very homogeneous sample in terms of age, gender (all female) and profession (all students). In order to still ensure sufficient variability in trait differences of emotional experience in daily life, the participants of this study were recruited in different steps using neuroticism as an indicator for trait emotional experience. As a first step, a sample of 268 female students from the University of Groningen and the Hanze University of Applied Sciences in Groningen completed the 12-item Neuroticism scale of the NEO Five-Factor Inventory (NEO-FFI, Hoekstra et al., 1996). From this sample, 75 students were selected using the 60th percentile score of the neuroticism scale (score = 31) of the NEO-FFI as criterion, randomly choosing 50 participants who scored above, and 25 who scored below this criterion. This selection procedure resulted in an approximately normal distribution of neuroticism scores (*M* = 133.84, SD = 21.33) as reassessed with the 48-item neuroticism scale of the Revised NEO Personality Inventory (NEO-PI-R; Hoekstra et al., 1996). Neuroticism is especially well suited as indicator and selection criterion in this study, since it is a well described stable trait difference in emotional experience as well as positively correlated with both indicators of emotion dynamics in daily life used in this study (i.e., NA-Inertia and root of the mean squared successive difference (RMSSD); Houben et al., 2015). All participants met the additional inclusion/exclusion criteria consisting of the absence of present or past psychiatric diagnosis, right handedness, not being under the influence of psychotropic medication and suitability to undergo an fMRI-scan (e.g., no metal implants, no claustrophobia etc.).

The resulting sample of 75 female students was then enrolled in the ESM part of the study. Seventy-one (95%) completed more than the a-priori defined cut-off point of responding to at least 60 measurement time points (85% of all measurement time points) during the ESM and participated in the fMRI part of the study (resulting in a mean response rate to ESM measurement time points of 93% in the final group of participants). From these, six participants were further excluded from analysis due to technical reasons or excessive motion artifacts (as further described in the “Analysis” section). As a result, the final sample consisted of 65 female students between 18 years and 25 years (*M* = 21, SD = 1.8). All participants were native Dutch speakers, had normal hearing, normal or corrected-to-normal vision, and gave written informed consent to participate in the study.

### Design

#### ESM

ESM measurements were programmed to take place five times a day at fixed time points with 3-h intervals adapted to the waking hours of the participant for a period of 14 consecutive days. At each measurement time point participants were alerted by a signal and presented with a short questionnaire through Personal Digital Assistants (PDAs; Myin-Germeys et al., 2009) or their own smartphones via a web-based application (ROQUA^1^). The ESM-Questionnaire included a list of six NA items (“upset,” “irritated,” “nervous,” “listless,” “down,” and “bored”), on the basis of which participants had to rate how strongly they experienced the specific emotion at the current moment, on a scale ranging from 1 (not at all) to 7 (very).

#### Social Feedback Task

The social feedback task (based on Davey et al., 2011) was designed to have participants receive fictive positive and negative social feedback from peers. In order to ensure that the participants perceived the feedback they received as coming from actual peers the task consisted of two steps, one outside and one inside the scanner.

For the introduction session outside the scanner each participant was asked to submit a neutral passport photo of herself as well as sort the pictures of faces of 36 unfamiliar same-aged peers into two groups of 18 pictures of peers they would like to work with on a new project and 18 pictures of peers they would not like to work with. The participants were told, that based on their initial impressions, pictures of faces would be selected and shown to them again during the fMRI experiment allowing to study how a first impression relates to the neural response on seeing faces a second time. Additionally, participants were told that their own face would be rated by their peers to select appropriate stimuli for their MRI sessions, and that after the study all pictures would be deleted.

Before the MRI session, 5–6 weeks after the introduction session, participants were reminded again that they would be presented with a subset of the faces they judged on first impression during the study’s introductory session for a second time during the MRI task. Additionally, participants were told that these faces, would be complemented by information about the willingness of this person to work with them on the new project. During the social feedback task itself, each participant passively viewed 72 faces (with neutral facial expression) for 4 s each (Inter Stimulus Interval: 3.5–4.5 s, Inter Onset Interval: 7.5–8.5 s). One second after the stimulus onset, the background of the picture turned into red (“negative,” meaning the other participant allegedly indicated they would not want to work with the participant), green (“positive,” meaning the other participant allegedly indicated they would like to work with the participant), or blue (“neutral, meaning that the other participant allegedly had not rated the participant’s picture”). The feedback was presented in six blocks, each consisting of four positive feedback, four negative feedback and four neutral feedback. Within the whole task, but not within one block, each face (always tight to a specific feedback) was shown twice.

After the fMRI-Session, the credibility of the fictive social feedback was checked by asking participants to rate how positive/negative (from −3 very unpleasant to 3 very pleasant) they experienced the positive/negative feedback as well as how tense (from 1 “not” to 7 “very”) they felt during the social feedback. Additionally, participants were asked to answer open questions, about how they felt about giving and receiving social feedback and if they had noted something particular about the task. All these measures pointed to a good credibility of the feedback with reportedly feeling moderately tense (*M* = 3.6, SD = 1.5) during the task as well as experiencing positive feedback as overall more pleasant (*M* = 1.6, SD = 0.9) and negative feedback as overall more unpleasant (*M* = −0.7, SD = 0.8). Additionally, in the open questions, only 6 of the 71 originally enrolled participants (8.5%) expressed doubts about the authenticity of the social feedback.

Debriefing followed a semi-standardized format in which participants were explained, that the photographs we used were not from actual participants in the study, but were taken from an existing set of photographs that are often used for research, that the feedback they received was generated by a computer and that no other research participant saw their photo. At the end of the debriefing every participant was encouraged to ask any question about the study and possible missing information about the study procedure.

### Analysis

#### ESM Data

A momentary NA scale was calculated for each subject at each time point by averaging the NA items for every rating-occasion. The resulting scale yielded a multilevel equivalent of Cronbach’s alpha (as proposed in Nezlek, 2017) of 0.98 indicating a good internal consistency.

From the momentary NA scale, two central parameters were obtained to capture the two main types of emotion dynamics: (1) the square RMSSD as an indicator for negative emotional instability; and (2) the autocorrelation (or rather, autoregressive effect) as a measurement of negative emotional inertia. These parameters were obtained within a multilevel analysis framework, taking into account the nested structure of the data as proposed in Jahng et al. (2008) (see also Koval et al., 2015; see Supplementary Material 1 for more information on the calculation of these parameters as well as Supplementary Material 2 for graphs depicting the example time-series and the distribution of the main ESM-parameters used in this study; further visualizations of Inertia and RMSSD can be found in Houben et al., 2015). In order to avoid taking into account the relation of the last and first time point of two consecutive days, between-day lags were omitted in the calculation of these scores. Finally, the average level of the NA scale was calculated per participant as a control variable in subsequent analyses.

#### fMRI Data

Brain imaging data were acquired using a 3.0 Tesla MRI scanner (Philips Medical Systems, Best, Netherlands) equipped with an 32-channel SENSE head coil. Functional images were acquired using a T2*-weighted echo-planar sequence with 37 axial slices recorded in descending manner (voxel size = 3.5 × 3.5 × 3.5 mm (42.875 mm^3^), TR = 2,000 ms, TE = 20 ms, FOV = 224 × 129.5 × 224 mm). To reduce artifacts from the nasal cavities, images were tilted 30° from the transverse plane of the anterior and posterior commissures. In addition, a shim box was placed onto orbitofrontal regions. High-resolution T1-weighted structural images were acquired containing 170 slices (voxel size = 1 × 1 × 1 mm, TR = 9 ms, TE = 8 ms, FOV = 232 × 170 × 256 mm).

#### Preprocessing

Preprocessing of the structural MRI data including registration to Talairach space, intensity normalization, removal of non-brain tissue was performed with Freesurfer^2^ (Fischl, 2012; Fischl et al., 2004). Functional Image preprocessing and analysis were performed using AFNI^3^ (Cox, 2012) within a framework of R software^4^ (Boubela et al., 2012). Standard preprocessing procedures included slice timing correction, co-registration and normalization into Talairach space. Images were smoothed using a 6-mm full-width half-maximum Gaussian kernel, and the signal time course was scaled to percentage signal change relative to the mean signal across time in each voxel. To correct for motion artifacts, volumes with excessive motion were censored (Euclidean norm > 0.3). Four subjects had more censored volumes than the pre-defined cut-off point of more than 40% of the acquired volumes and were excluded from the analysis.

#### Subject-Level Analysis

Single subject BOLD responses at stimulus onset were modeled using GLM (3dDeconvolve). The hemodynamic response to each event type (positive, negative, neutral) was modeled with a delta function at the feedback onset and convolved with the gamma-variate hemodynamic response function. Additionally, contrast maps of the event types were created (positive-neutral, negative-neutral). Only the valence of the feedback was considered in this analysis (see Supplementary Material 3 for further analysis also including the decision of the participant). Nuisance regressors included low frequency drift (linear, quadratic and cubic) and motion (L/R, A/P, S/I, roll, pitch, yaw, and their derivatives).

#### Group-Level Analysis

On the group level the event-contrast maps (negative-neutral, positive-neutral) were analyzed in a voxel-wise analysis using an ANCOVA-like design based on Omnibus F-Tests (3dMVM; Chen et al., 2014). The model included valence (negative, positive) as within-subject variables as well as NA-RMSSD and NA-inertia scores as obtained from the multilevel models as between-subject variables. Additionally, the interaction effects between RMSSD and valence, and inertia and valence were included. Average NA as well as the interaction term for average NA and valence were introduced as variable of no interest in order to correct for possible confounding effects (Jahng et al., 2008). Also testing for the main-task effects of positive as well as negative feedback, a contrast for the main effect of positive as well as a contrast for the main effect of negative valence were included in the analysis as planed comparisons.

In order to find significant activation-clusters the resulting parametric maps were thresholded at a voxel-level *p*-value of 0.005. Subsequently, in order to determine the significance of the resulting clusters, Monte-Carlo simulations were used to calculate the likelihood of observing a cluster with a certain size in white noise using the newest version of 3dClustSim (Cox et al., 2017), resulting into a lower limit of 16 Voxels (686 mm^3^) for a corrected significance level of *p* < 0.05.

For subsequent *post hoc* tests individual median parameters of the contrasts between positive and neutral as well as negative and neutral feedback were extracted from all significant clusters. Further evaluating the effects found in the main analysis, cluster-wise random-intercept multilevel models (using the nlme-package in R; Pinheiro et al., 2018) were fitted on the parameters, including both responses to positive and negative social feedback. Estimating valence-general effects, the models included NA-RMSSD, NA-Inertia as well as mean NA. For the estimation of valence specific effects, the interaction effect between valence of the feedback and NA-RMSSD as well as NA-Inertia was additionally included into the model. In order to further investigate, whether the association between those parameters and the ESM-measures were unique for one of the ESM-measures (see also Nieuwenhuis et al., 2011), the effects of NA-Inertia and NA-RMSSD were compared directly using the linearHypothese() function of the car-package (Fox and Weisberg, 2011) in R.

## Results

### ESM-Results

The mean NA rating across participants was 2.20 (SD = 0.85) and the mean of parameters for NA-RMSSD and NA-Inertia were 0.63 (SD = 0.20) and 0.32 (SD = 0.12), respectively. Both inertia [*r* = 0.58, 95% CI (0.40; 0.72), *t*_(62)_ = 5.9, *p* < 0.001] as well as RMSSD [*r* = 0.46, 95% CI (0.25; 0.63), *t*_(62)_ = 4.3, *p* < 0.001] were positively correlated to mean NA scores but not to each other. Neither taking the average NA into account [*r* = −0.14, 95% CI (−0.37; 0.11), *t*_(62)_ = −1.1, *p* = 0.29] or not into account [*r* = 0.07, 95% CI (−0.16; 0.30), *t*_(62)_ = 0.6, *p* = 0.54] yielded a significant correlation between NA-Inertia and NA-RMSSD.

### Imaging Results

#### Task Results

Negative (i.e., negative feedback vs. neutral feedback) as well as positive (i.e., positive feedback vs. neutral feedback) social feedback were both connected to a widespread pattern of activations including anterior midline structures (mPFC, ACC), salience regions (anterior insula, ventral striatum), medial temporal lobe structures (including the hippocampal formation and the mediotemporal gyrus) as well as the bilateral dorsal lPFC, inferior frontal gyrus and visual areas of the occipital lobe (see Supplementary Tables S3,S4 in the Supplementary Material 4 for the full results of this analysis; see also the results of an contrast between negative and positive social feedback as well as graphs depicting the unique and overlapping activations connected to positive and negative feedback in Supplementary Material 4).

#### Relations With Emotion Dynamics

**Figure 1:**
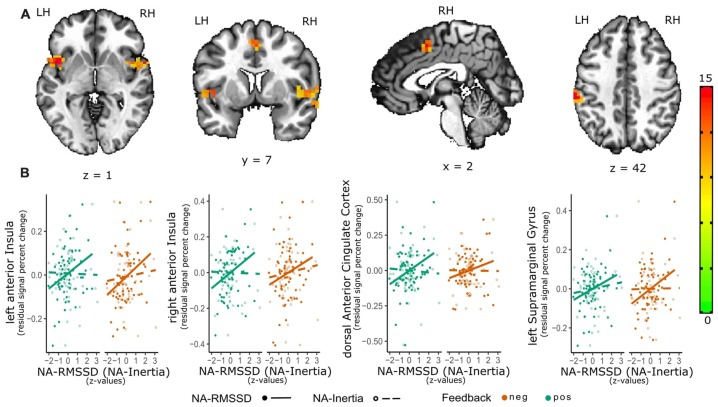
Relation of emotional instability of negative affect (NA-RMSSD) and the neural response to social feedback. The activation maps **(A)** depict the significant clusters (as estimated with 3dClustSim) of the valence-general effect in the bilateral anterior Insula (aIns), dorsal anterior cingulate cortex (dACC) as well as supramarginal gyrus (SMG). In order to further visualize the association of the activity in the single clusters with NA-RMSSD, additional scatterplots **(B)** are shown in the lower panel of the graph. For these scatterplots individual median percent signal change by the positive-neutral feedback (green) as well as the negative-neutral feedback (red) were extracted from these clusters and plotted against individual RMSSD scores (full circles; solid line). In order to show the contrast of the relation between activity in the clusters and NA-RMSSD to the relation with NA-Inertia, also the association of the activity in these clusters with Inertia (dashed line) and individual Inertia scores (empty circles) are depicted in the scatterplots. Facilitating the depiction of this contrast NA-Inertia as well as NA-RMSSD are z-transformed. Furthermore, to remove possible confounds of a relation of activation in these clusters and mean experienced NA, individual differences in average NA have been regressed out of the percent signal change values.

Individual differences in NA instability (RMSSD) were related to activation in the right [peak = (47 7 3); *F*_(1,60)_ = 19.7, 56 voxels, cluster-wise *p* < 0.01] as well as left anterior insula [aIns; peak = (−45 7 1), *F*_(1,60)_ = 19.8, 27 voxels, cluster-wise *p* < 0.01], dACC [peak = (1 5 45), *F*_(1,60)_ = 15.1, 19 voxels, cluster-wise *p* < 0.05] and left supramarginal gyrus [SMG; BA 40; peak = (−56 −28 40), *F*_(1,60)_ = 21.1, 27 voxels, cluster-wise *p* < 0.01; see Figure 1]. There were no significant interaction effects for RMSSD and valence of the social feedback. *Post hoc* analysis of the parameters of the positive vs. neutral as well as negative vs. neutral feedback contrast and RMSSD showed that activity in the left aIns (*β* = 0.064 (0.014), *t*_(60)_ = 4.39, *p* < 0.001) as well as right aIns (*β* = 0.066 (0.018), *t*_(60)_ = 3.75, *p* < 0.001) were positively related to RMSSD as well as this effect being significantly or trend-wise significantly (left aIns: $X_{\left(1\right)}^{2}$ = 5.04, *p* = 0.024; right aIns $X_{\left(1\right)}^{2}$ = 3.23, *p* = 0.072) different from the association of NA-Inertia and activity within these clusters [left aIns: *β* = 0.027 (0.014); right aIns: *β* = 0.030 (0.017)]. Similarly also the activation within the dACC cluster (*β* = 0.059 (0.015), *t*_(60)_ = 3.79, *p* < 0.001) and IPL-cluster (*β* = 0.057 (0.013), *t*_(60)_ = 4.26, *p* < 0.001) were positively associated with NA-RMSSD. The association of NA-RMSSD and activity in the dACC cluster was additionally significantly different ($X_{\left(1\right)}^{2}$ = 6.42, *p* = 0.011) from the activity within this cluster and inertia (*β* = 0.014 (0.015) and the association with the activity of the IPL-cluster trend-wise significantly different ($X_{\left(1\right)}^{2}$ = 3.72, *p* = 0.054) from the association with NA-Inertia (*β* = 0.028 (0.013) ;see also Figure 1).

**Figure 2:**
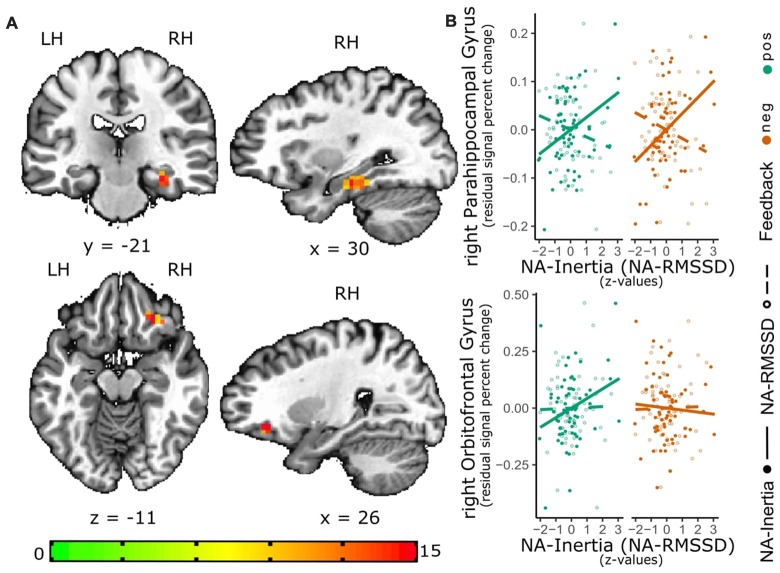
Relation of NA-Inertia and the neural response to positive and negative social feedback. The activation maps **(A)** depict the significant cluster (as estimated with 3dClustSim) of the valence-general effect in the right parahippocampal gyrus (PHG) as well as the significant cluster for the interaction effect of valence and NA inertia in the right lateral orbitofrontal cortex (lOFC). In order to further visualize these effects, scatterplots **(B)** of this associations have been added to the graph. For these scatterplots individual median percent signal change by the positive-neutral feedback (green) as well as the negative-neutral feedback (red) were extracted from these clustersand plotted against individual inertia scores (full circles; solid line). In order to show the contrast of the relation between activity in the clusters and NA-Inertia to the relation with NA-RMSSD, also the association of the activity in these clusters with RMSSD (dashed line) and individual RMSSD scores (empty circles) are depicted in the scatterplots. Facilitating the depiction of this contrast NA-Inertia as well as NA-RMSSD are z-transformed. Furthermore, to remove possible confounds of a relation of activation in these clusters and mean experienced NA, individual differences in average NA have been regressed out of the percent signal change values.

Inertia of NA was found to be correlated with the right parahippocampal gyrus [PHG; peak = (30 −22 −14), *F*_(1,60)_ = 14.6, 24 voxels, cluster-wise *p* < 0.01]. Additionally, there was a significant interaction effect between inertia and valence in the right lateral orbitofrontal cortex [lOFC; peak = (26 31 −11), *F*_(1,60)_ = 24.5, 16 voxels, cluster-wise *p* < 0.05]. *Post hoc* analysis showed that the activity of the right PHG (*β* = 0.037 (0.009), *t*_(60)_ = 4.12, *p* < 0.001) was positively correlated with inertia, while the interaction effect between NA-Inertia and valence of the social feedback indicated an positive association between NA-Inertia and the lOFC during negative but not positive social feedback (*β* = 0.052 (0.014), *t*_(61)_ = 3.53, *p* < 0.001, see also Figure 2). Additionally, the association of NA-Inertia and activity within the right PHG cluster was significantly different ($X_{\left(1\right)}^{2}$ = 21.0, *p* < 0.001) from the association with RMSSD [*β* = −0.01 (0.009)]. Similarly, the interaction effect between NA-Inertia and valence of the social feedback in the right lOFC was significantly different ($X_{\left(1\right)}^{2}$ = 6.27, *p* = 0.012) from the interaction of RMSSD and social feedback-valence [*β* = 0.001 (0.014)]. All cluster peaks are reported in TLRC space (LPI).

Anatomical labels were assigned to the clusters using the TT Daemon-Atlas^5^ as well as visual inspection and additional sources as prior studies. For instance, the peaks of the clusters in the anterior insula where labeled “insula” by the TT Daemon-Atlas as well as close to the peaks of clusters, identified as anterior insula regions of the SN in prior studies using independent component analysis (peaks within 6 mm; see for example Doll, 2013; White et al., 2013), indicating the label “anterior insula”.

As outlined in the analysis section, the analysis reported here was only considering the feedback of the peer to the participant as determining the valence of the social feedback. However, it is possible that also the evaluation of the peer (“I want to work with her” or “I do not want to work with her”) before the scan session by the participant might have influenced the effect of the social feedback. In order to visualize such potential differences, we are also reporting an additional analysis considering this variable in the Supplementary Material 3.

## Discussion

In the present study, we investigated how individual differences in the tendency for negative emotional experiences to be unstable or inert in real life are associated with neural responses to positive and negative socio-emotional events inside the scanner.

In line with our general hypotheses, our findings are indicating that trait differences of the two studied emotion dynamics are uncorrelated to each other and related to different aspects of the emotional response. Instability of negative emotional experience (RMSSD) was found to be correlated to increased activity in the bilateral anterior insula (aIns) and dACC as well as left SMG in response to negative and positive feedback. Inertia of NA was found to be connected to increased activity of the right PHG to positive and negative feedback as well as right lOFC in response to negative feedback.

Interestingly, these regions are part of different functional networks in the brain. The bilateral aIns and dACC exhibit strong structural (van den Heuvel et al., 2009) as well as functional connectivity, building the core of an intrinsic network; the SN. This network is considered to play a key role in the detection and processing of subjective salience, activating in response to emotional arousing information (Seeley et al., 2007; Uddin, 2015). Also, the SMG has been shown to have functional connections to the SN (Mars et al., 2012) and is especially found to activate together with this network in socio-emotional contexts (Kanske et al., 2015). In contrast, the OFC (see Wilson et al., 2014; Schuck et al., 2016) as well as the PHG (see Aminoff et al., 2013) have been implicated in representing different domains of contextual information. These representations might be complimentary, with input of the hippocampal formation having been proposed to be essential forming a complex associative cognitive map in the OFC, defining the current task- or state-space. (Alm et al., 2015; see also Stalnaker et al., 2015; Wikenheiser and Schoenbaum, 2016).

Our findings are partly in line with previous results from behavioral and resting state studies investigating individual differences in intrinsic functional connectivity and emotion dynamics in everyday life. Especially the obtained association of emotional instability and increased responses in the SN is in line with previous research finding a relation between emotional instability and altered functional connectivity of the SN at rest in remitted depressed subjects (Servaas et al., 2017), as well as research finding a relation between emotional instability and increased emotional reactivity in daily life (Thompson et al., 2012). The association of inertia with the OFC as well as PHG is less consistent with resting state findings, suggesting an association between inertia and the lPFC (Waugh et al., 2017). In this context it should be noted, that the analysis described by Waugh et al. (2017) focused on altered changes of intrinsic functional connectivity after an emotional task, what might rather reflect differences in the engagement and processing of the task, than individual differences in the organization of resting state networks or altered responses to emotional information.

In general, the neural correlates of NA instability as well as the neural correlates of NA inertia were similar during positive and negative feedback. Only activation of the lOFC showed a significant interaction effect, with activity in this region only being associated with inertia during negative feedback. This valence specific effect is in line with studies showing that lOFC is especially sensitive to negative emotion and punishment (Kringelbach and Rolls, 2004).

An important strength of the present study lies in relating brain processes during emotional events in the scanner to the dynamics of people’s feelings throughout their everyday lives. As such, it contributes to demonstrating the explanatory role of neuroimaging research in highly standardized settings for everyday thoughts, feelings, and behavior. The current findings are extending our knowledge about the neural mechanisms underlying emotion dynamics by showing that trait differences are not only reflected in the organization of resting state networks but also the processing of socio-emotional information. Additionally, consistent with the proposal of Cunningham et al. (2013), we find that emotion dynamics, reflecting a tendency towards sustained emotional experience, compared with emotion dynamics, reflecting a tendency towards changes in the emotional experience, are linked to different neural aspects of the socio-emotional response being grounded in different functional networks. It is important to point out that we focus in this study on complex socio-emotional events, since functioning in socio-emotional contexts is, as discussed before, related to emotion dynamics as well as especially important for the development and maintenance of affective disorders. Not additionally involving non-social emotional events, however leaves, as a limitation, unclear if the described relationship is specific for such a context. An additional important limitation is that the study population was restricted to young and healthy female participants and it is unclear to extend the results would generalize to other populations as males or older people.

This is, to our knowledge, the first study investigating the association between neural responses to emotional events in the scanner and aspects of emotional dynamics in everyday life. Our findings are generally supporting the hypothesis that different aspects of the neural response to socio-emotional events can be connected to different aspects of emotion dynamics in daily-life. Furthermore, the results are pointing towards these aspects representing different functional domains. While the regions of the salience system and specifically the insula are connected with detecting and initiating reactions towards important events, the OFC and PHG are ideally suited to contextually integrate such events. Testing such a hypothesis by directly manipulating those functional aspects could be a very important future direction helping to understanding the neural mechanisms of emotion dynamics as well as, more general the neural correlates of emotional experience. Important next steps would also include to test, whether such a relationship is specific to socio-emotional contexts or generalize to emotional processes as a whole.

## Ethics Statement

This study was carried out in accordance with the recommendations of the Medical Ethical Committee of the University Medical Center Groningen with written informed consent from all subjects. All subjects gave written informed consent in accordance with the Declaration of Helsinki. The protocol was approved by the Medical Ethical Committee of the University Medical Center Groningen.

## Author Contributions

JB and AO designed the study and collected the data. JP, PK, PF and PV analyzed the data. JP, PK and PV wrote the manuscript. All authors discussed the findings as well as reviewed and revised the manuscript.

## Conflict of Interest Statement

The authors declare that the research was conducted in the absence of any commercial or financial relationships that could be construed as a potential conflict of interest.

## Footnotes

^1^www.roqua.nl

^2^http://surfer.nmr.mgh.harvard.edu

^3^http://afni.nimh.nih.gov/afni

^4^https://cran.r-project.org/

^5^https://afni.nimh.nih.gov/AFNIAtlases
