# Supplementary material for: Different Aspects of the Neural Response to Socio-Emotional Events Are Related to Instability and Inertia of Emotional Experience in Daily Life: An fMRI-ESM Study
Source: Front Hum Neurosci. 2018 Dec 11;12:501. doi: 10.3389/fnhum.2018.00501 (PMC6297363; doi:10.3389/fnhum.2018.00501)
Supplement: Supplementary file 1 [file Data_Sheet_1.docx]

Supplementary Material

**Different aspects of the neural response to socio-emotional events are related to instability and inertia of emotional experience in daily life: An fMRI-ESM Study**

**Julian Provenzano^1*^ , Jojanneke A. Bastiaansen^2,3^, Philippe Verduyn^4^, Albertine J. Oldehinkel^2^, Philippe Fossati^5,6^, Peter Kuppens^1^**

***Correspondence:** Julian Provenzano: Julian.Provenzano@kuleuven.be

**Supplementary Material 1:** Estimation of ESM-paramters.

NA-RMSSD and NA-Inertia represent two different relationships between lagged-measures, i.e. relation between the measurement of negative affect NA_t_ at a specific time-point and the measurement NA_t-1_ at the previous time point. Consequently, these parameters can be seen as a relationship between the negative affect time series (NA) and the corresponding lagged NA time series. The RMSSD reflects the average distance between two consecutive measures, therefore indicating the level of emotional instability. The autocorrelation reflects the prediction of the time series by the lagged version of itself, therefore indicating the extent to which emotional states carry-over across time, reflecting inertia.

Due to the nested structure of the data (with time points nested within individuals), both RMSSD and inertia of NA were modeled in a multilevel or mixed-model approach. In these analyses, between-day lags are omitted to avoid the inclusion of day-to-day carry over effects.

**NA RMSSD.** Following Jahng, Wood, & Thrull (2008), person-specific NA-RMSSD scores were estimated using a random-intercept model on a variable that was calculated as the square root of the squared successive difference (i.e. the absolute difference) between two consecutive time points (omitting over-day lags):

$$|{NA}_{(t-1)j}-{NA}_{tj}|= \beta_{0}+u_{0j}+e_{j}$$

orLevel- 1: $|{NA}_{(t-1)j}-{NA}_{tj}|= \beta_{0 j}+e_{j}$

Level- 2:$\beta_{0j}= \beta_{0}+ u_{0j}$ (random intercept term)

In this equation NA_tj_ represents the negative affect of a person j at time point t, β0 represents the overall intercept, or the average square root of squared (i.e. absolute) difference between consecutive time points. u_0j_ represents the person-specific deviations from this overall intercept (with average = 0), reflecting individual differences in the average distance between consecutive measurements.

**NA Inertia.** Person-specific NA-inertia scores were estimated using a first-order autoregressive model (Butler, 2011; Gottmann et al., 2005; Kuppens, Allen, et al., 2010; Suls et al., 1998) that can be seen as a special case of a random-intercept random-slope multilevel model:

$${NA}_{tj}= \beta_{0}+ \beta_{1}{NA}_{\left( t-1 \right)j}+u_{0j}+ u_{1j}{NA}_{\left( t-1 \right)j}+e_{tj}$$

or

Level- 1 ${NA}_{tj}= \beta_{0j}+ \beta_{1j}{NA}_{\left( t-1 \right)j}+e_{ij}$

Level -2 $\beta_{0j}= \beta_{0}+ u_{0j}$ (random-intercept term)

$\beta_{1j}= \beta_{1}+ u_{1j}$ (random-slope term)

where NA_tj_ represents the negative affect of a person *j* at a specific occasion *t* and NA_(t-1)j_ the corresponding lagged predictor. In this model the intercept (β_0j_ ) as well as the slope (β_1j_ ) are consisting of a fixed (grand mean) part (β_0_ as well as β_1_) but are also allowed to vary over subjects by the subject-specific parameters u_0j_ and u_1j_. Accordingly β_1j_  represents the subject-specific slopes between negative affect on occasion *t* and the negative affect at the lagged occasion *t-1*. Therefore, this term can also be seen as a subject-specific autoregressive term representing the autocorrelation of the NA-scale, or negative affect inertia.

**Supplementary Material 2:** Visualization of ESM-data as well as ESM-parameters.

**Figure S1: Visualization of NA-RMSSD.** In order to visualize RMSSD in the time-series of negative affect-ratings in the ESM-part of the study, the first seven days of two example participants are shown. Example Subject A scored high on RMSSD (NA-RMSSD = 1.03; NA-Inertia = 0.13, mean negative affect = 3.23) while example subject B scored low on RMSSD (NA-RMSSD = 0.30, NA-Inertia = 0.27, mean negative affect = 1.19) compared to the study-sample (M = 0.63, SD = 0.20).

**Figure S2: Visualization of NA-Inertia.** In order to visualize inertia (i.e. autocorrelation) in the time-series of negative affect-ratings in the ESM-part of the study, the first seven days of two example participants are shown. Example Subject A scored high on inertia (NA-Inertia = 0.62; RMSSD = 1.01, mean negative affect = 3.20) while example subject B scored low on inertia (NA-Inertia = 0.08, RMSSD = 0.67, mean negative affect = 2.07) compared to the study-sample (M = 0.32, SD = 0.12).

**Figure S3:**

**Figure S3: Histograms of the main ESM-parameters.** Distribution of mean negative affect, NA-Inertia and NA-RMSSD parameters across all participants included into the study.

**Supplementary Material 3:**

Before the social feedback tasks, participants themselves indicated whether they wanted to work on a project with the people depicted in the pictures, that would later be used in the social feedback task. Consequently, the experienced valence of the feedback might have been modulated by how the peer giving the feedback was perceived by the participant (i.e. negative feedback of a peer rated positive might be more negative as negative feedback of a peer rated negatively).

To visualize how such effects might be reflected in our results, we performed additional analyses. In these analyses, six instead of three events were modeled, not only taking into account the feedback of the peer (positive, negative or neutral), but also if the shown peer was rated before the session as positive (“I want to work with her”) or negative (“I don’t want to work with her”) by the participant. Also here, subject specific contrast maps were calculated for each emotional feedback, contrasting the emotional feedback with the corresponding neutral feedback (i.e. peer given feedback being positive rated before the session and peer giving negative feedback vs. peer given feedback being positive rated before the session and giving neutral feedback etc.). Regions of interests were derived from the main results, using the significant clusters in the dorsal anterior cingulate (dACC), right and left anterior Insula (aIns), supramarginal gyrus (SMG), right lateral orbitofrontal cortex (lOFC), right parahippocampal gyrus (PHG) as masks to extract the median parameters for every contrast map (NegPos, PosPos, PosNeg, NegNeg) for every subject. In order to estimate main effects of how the peer was rated by the participant on activations within the ROIs for a specific feedback of the peer, at first, t-tests, comparing different ratings before the session on responses during negative or positive feedback, were calculated. Subsequently simple regressions including NA-RMSSD and NA-inertia, controlling for mean NA were calculated on the extracted parameters of every contrast map. Regressions as well as t-test were conducted using R (<http://cranr-project.or/>). The sole purpose of this analysis was to visualize the consistency of the effects over the additional conditions of how the subject rated the picture of the peer, she was getting feedback from. It has to be pointed out here, that taking into account this additional conditions limits the number of stimuli per subject and condition and, as a result, the analysis might be severely underpowered and the single results have to be considered with caution. However we still belief that the profile of the effects over these condition might be meaningful additional information to the findings presented in the main analysis. Reflecting this purely visualizing and post-hoc character of this analysis none of the effects were corrected for multiple comparisons.

**Results.** Investigating the influence of the previous rating of the peer on neural responses of the ROIs during her positive or negative feedback, only the activity of the right anterior insula during positive feedback was significantly modulated by the previous rating with increased activation when the participant had rated the peer negatively (M_diff_ = 0.08, t = 3.54, p < 0.01). All other comparisons did not yield any significant results (all p > 0.23).

For the condition of receiving negative feedback from peers rated negatively before, a positive relationship between RMSSD and activity in the left (β = 0.35, t = 2.43, p < 0.05) and right (β = 0.27, t = 2.61, p < 0.05) aIns as well as a trend-wise significant positive relationship for RMSSD and the dACC (β = 0.27, t = 1.82, p < 0.1) and SMG (β = 0.18, t = 1.85, p < 0.1) was found. In the same condition inertia showed a trend-wise significant positively relationship to activity in the right lOFC (β = 0.59, t = 1.87, p < 0.1) and right PHG (β = 0.35, t = 1.75, p < 0.1).

In the condition of receiving negative feedback from a peer rated positively, RMSSD was positively related to activity in the right aIns (β = 0.27, t = 2.32, p < 0.05) as well as trend wise positively related to activity in the left aIns (β = 0.24, t = 1.73, p < 0.1) as well as SMG (β = 0.21, t = 1.85, p < 0.1). For inertia none of the relationship reached significance (all p > 0.58).

Receiving positive feedback of a peer rated negatively, a positive relationship between RMSSD and responses in the dACC (β = 0.56, t = 3.19, p < 0.01), left (β = 0.35, t = 2.47, p < 0.05) and right ( β = 0.27, t = 2.6, p < 0.05) aIns as well as SMG (β = 0.37, t = 2.7, p < 0.01) were found. With inertia there was a significant relationship with responses in the right PHG (β = 0.56, t = 3.12, p < 0.01) but not right lOFC (β = -0.61, t = -1.59, p > 0.1).

For receiving positive feedback from peers that have been rated positively before none of the relationships with inertia or RMSSD reached significance (all p > 0.2).

**Table S1:** Effects and standard deviationsof RMSSD for extracted ROIs for every combination of rating of the participant (before the scanning) and feedback of the peer (during the task)

| **Rating** | **Feedback** | **dACC** | **Right aIns** | **Left aIns** | **Left SMG** |
| --- | --- | --- | --- | --- | --- |
| positive | negative | 0.05 (0.14) | 0.27*(0.12) | 0.24’ (0.14) | 0.21’ (0.11) |
| negative | negative | 0.27’(0.15) | 0.27* (0.10) | 0.31* (0.13) | 0.18’ (0.11) |
| positive | positive | 0.00 (0.14) | 0.04 (0.12) | 0.14 (0.13) | 0.05 (0.11) |
| negative | positive | 0.56**(0.18) | 0.27*(0.13) | 0.35*(0.14) | 0.37** (0.14) |

‘ 0.1 * 0.05 ** 0.01

**Table S2:** Effects and standard deviations of inertia for extracted ROIs for every combination of rating of the participant (before the scanning) and feedback of the peer (during the task)

| **Rating** | **Feedback** | **Right lOFC** | **Right PHG** |
| --- | --- | --- | --- |
| positive | negative | 0.19 (0.46) | 0.09 (0.15) |
| negative | Negative | 0.59’ (0.32) | 0.34’ (0.19) |
| positive | Positive | -0.10 (0.73) | 0.22(0.17) |
| negative | Positive | -0.61 (0.38) | 0.56**(0.18) |

‘ 0.1 * 0.05 ** 0.01

**Supplementary Material 4**: Additional information regarding main task effects of the social feedback task.

**Table S3**: Significant clusters and cluster-peak TLRC Coordinates (LPI) of the contrast between negative social Feedback and neutral Feedback as well as the size of the clusters and the corrected significance levels of these clusters (as estimated with 3dClustSim)

| **Regions** | **x** | **y** | **z** | **Voxels** | **t-Value** | **p** |
| --- | --- | --- | --- | --- | --- | --- |
| Occipital Lobe | 19 | -60 | 6 | 2189 | -6.9 | < 0.001 |
| mPFC/ACC/Superior Frontal Gyrus | -5 | 41 | 20 | 641 | 5 | < 0.001 |
| right anterior Insula/ right Inferior Frontal Gyrus/ right Middle Temporal Gyrus | 43 | 18 | -3 | 318 | 5.8 | < 0.001 |
| left anterior Insula/ left Inferior Frontal Gyrus/ left Middle Temporal Gyrus | -37 | 17 | -6 | 236 | 5.8 | < 0.001 |
| left dlPFC | -23 | 13 | 41 | 37 | -4.3 | < 0.01 |
| right dlPFC | 23 | -1 | 45 | 68 | -3.4 | < 0.001 |
| left ventral Striatum  right ventral Striatum | -12  9 | 9  20 | 7  7 | 55  38 | 4.2  5.2 | < 0.001  < 0.01 |
| left Inferior Parietal Lobule | -29 | -32 | 38 | 35 | -3.4 | < 0.01 |
| left Parahippocampal Gyrus | -23 | -22 | -4 | 22 | -4.4 | < 0.05 |
|  |  |  |  |  |  |  |
|  |  |  |  |  |  |  |

**Table S4**: Significant clusters and cluster-peak TLRC Coordinates (LPI) of the contrast between positive social Feedback and neutral Feedback as well as the size of the clusters and the corrected significance levels of these clusters (as estimated with 3dClustSim)

| **Regions** | **x** | **y** | **z** | **t-Values** | **Voxels** | **p** |
| --- | --- | --- | --- | --- | --- | --- |
| Occipital Lobe / left Parahippocampal Gyrus | -12 | 64 | 10 | -6 | 1280 | < 0.001 |
| mPFC/ACC | 2 | -38 | 6 | 4.6 | 248 | < 0.001 |
| right anterior Insula | -30 | -20 | -4 | 3.9 | 62 | < 0.001 |
| left Inferior Frontal Gyrus | 30 | -20 | -11 | 3.3 | 27 | < 0.01 |
| left dlPFC | 20 | -20 | 38 | -3.7 | 57 | < 0.001 |
| right dlPFC  right Precuneus | -30  -12 | -24  39 | 34  45 | -3.9  -3 | 48  57 | < 0.01  <0.001 |
| left/ right ventral Striatum | 5 | -6 | -1 | 3.9 | 54 | < 0.001 |
| left Superior Temporal Gyrus | 58 | 32 | 10 | -3.7 | 71 | < 0.001 |
| right Superior Temporal Gyrus | -37 | 50 | 17 | -4,2 | 37 | < 0.01 |
|  |  |  |  |  |  |  |

**Table S5:** Significant clusters and cluster-peak TLRC Coordinates (LPI) of the contrats bewteen negative feedback and positive feedback as well as the size of the clusters and the corrected significance levels of these clusters (as estimated with 3dClustSim)

| **Regions** | **x** | **y** | **z** | **t-Value** | **Voxels** | **p** |
| --- | --- | --- | --- | --- | --- | --- |
| medial Occipital Lobe | 4 | -79 | 4 | 5 | 94 | < 0.001 |
| left Inferior Frontal Gyrus | -50 | 21 | 13 | 4.1 | 61 | < 0.001 |
| right Superior Temporal Gyrus | 47 | -28 | -1 | 4.4 | 60 | < 0.001 |
| Superior Frontal Gyrus | 3 | 27 | 51 | 2.9 | 43 | < 0.01 |
| right Inferior Frontal Gyrus | 47 | 25 | 3 | 4.2 | 39 | < 0.001 |
| left Middle Temporal Gyrus | -51 | 4 | -12 | 4.3 | 36 | < 0.01 |
| right Middle Temporal Gyrus | 50 | 6 | -17 | 3.6 | 21 | < 0.05 |
|  |  |  |  |  |  |  |

**Figure S4:** significant clusters of the negative (compared to neutral) social feedback (red) as well as positive (compared to neutral) social feedback (green) as well as the overlap between both (blue). Clusters with increased %-signal change of emotional compared to neutral feedback are depicted on the right while clusters with decreased %-signal change of emotional compared to neutral feedback are depicted on the left side.
